# Supplementary material for: The Ratio of RAC1B to RAC1 Expression in Breast Cancer Cell Lines as a Determinant of Epithelial/Mesenchymal Differentiation and Migratory Potential
Source: Cells. 2021 Feb 8;10(2):351. doi: 10.3390/cells10020351 (PMC7915250; doi:10.3390/cells10020351)
Supplement: Supplementary file 1 [file cells-10-00351-s001.pdf]

Figure S1

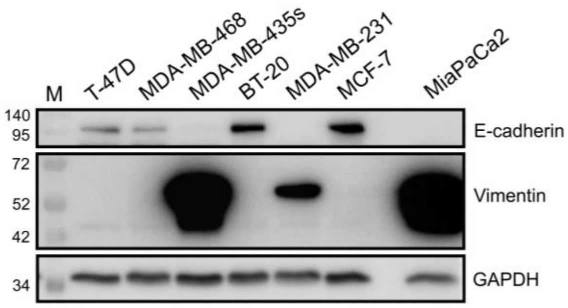

Figure S1. E-cadherin and vimentin expression in the same panel of BC cell lines shown in Figure 1. The pancreatic cancer cell line, MiaPaCa2, was used as a positive control for expression of vimentin [1]. Cells were grown to approx. 80% confluence, lysed and processed for immunoblot analysis of E-cadherin, vimentin, and GAPDH as a loading control. Data are representative of three samples for each cell line taken at different times during continuous culture. M, molecular weight marker. The numbers to the left denote the molecular masses in kDa.

Figure S2

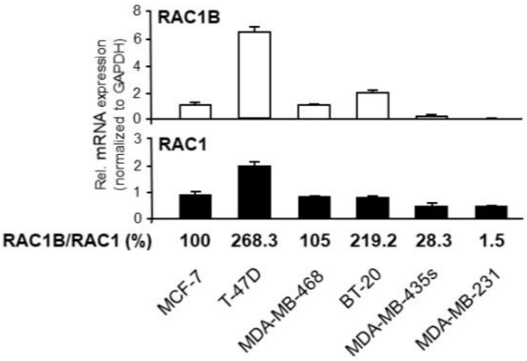

Figure S2. RAC1B and RAC1 mRNA expression in the same panel of BC cell lines shown in Figure 1. Cells were grown to approx. 80% confluence, lysed and processed for RNA isolation and qPCR for RAC1B (upper graph) or RAC1 (lower graph), and GAPDH as an internal control. Data represent the combined results of three samples for each cell line taken at different times during continuous culture (mean ± SD, n = 3). The ratios of RAC1B to RAC1 (RAC1B/RAC1) for each cell line is given below the RAC1 data and are expressed relative to the ratio in MCF-7 cells set arbitrarily to 100%.

1. Luley, K.B.; Biedermann, S.B.; Künstner, A.; Busch, H.; Franzenburg, S.; Schrader, J.; Grabowski, P.; Wellner, U.F.; Keck, T.; Brabant, G.; et al. A Comprehensive Molecular Characterization of the Pancreatic Neuroendocrine Tumor Cell Lines BON-1 and QGP-1. *Cancers* **2020**, *12*, 691.
